# Products With High Purchase Frequency Require Greater Inhibitory Control: An Event-Related Potential Study

**DOI:** 10.3389/fpsyg.2021.727040

**Published:** 2021-09-20

**Authors:** Koki Tsuji, Midori Shibata, Yuri Terasawa, Satoshi Umeda

**Affiliations:** ^1^Keio Global Research Institute, Keio University, Tokyo, Japan; ^2^Department of Psychology, Keio University, Tokyo, Japan

**Keywords:** Go/No-go task, cognitive control, response history, event-related potentials, N2 component

## Abstract

One’s past behavior influences their present behavior. The effects of such response history have often been tested using response inhibition tasks. Since previous studies have highlighted the effect of immediate action history formed directly before the subsequent response in a laboratory environment, we aimed to elucidate the longer-term effects of response history, using repetitive and habitual consumer behavior in daily life as the response history. We used event-related potentials recorded in a Go/No-go task to investigate brain activity related to inhibitory control, hypothesizing that stimuli with a high frequency of choice in everyday life would elicit stronger inhibition-related activity, that is, the No-go-N2 component. Participants were asked to evaluate the frequency of purchase and use of some products, such as food and drink or social networking services (SNS) in everyday situations. Images of each product were assigned as stimuli in the Go and No-go trials according to the frequency of choice. The results showed that frequently purchased No-go stimuli yielded a larger amplitude of the No-go-N2 component and a negative shift between 200 and 300ms after the presentation of No-go stimuli. The results suggest that frequently chosen products evoke stronger inhibition conflicts and require greater cognitive control to withhold a response. Our findings showed that repeated purchase behavior in daily life forms a response history and has a long-term influence on the inhibition of even simple approaching behaviors, such as button pressing.

## Introduction

One’s present behavior is influenced by their past behavior. There is a growing body of literature indicating that past responses to a stimulus influence future responses to that stimulus (Fecteau and Munoz, 2003; Freitas et al., 2007; Manoach et al., 2007). Previous studies focusing on the effect of response history on pro-saccade and anti-saccade tasks showed that previous saccades influence subsequent saccade latency (Cherkasova et al., 2002; Fecteau et al., 2004; Barton et al., 2006; Manoach et al., 2007; Reuter et al., 2019), thereby indicating that inhibitory control of the response system was modulated by a previous trial, that is, action history (for review, Fecteau and Munoz, 2003).

Studies on reaction history are not limited to saccadic control. Previous studies have also examined the effect of trial history in motor learning Witney et al. (2001), action choices (Valyear et al., 2019), and attentional orientation (Ciardo et al., 2019). In the research stream of cognitive control and response inhibition, several earlier studies using the Go/No-go task have revealed the effect of response history on subsequent response suppression. The more consecutively Go trials followed, the more likely it was to cause an error response in subsequent No-go trials (Durston et al., 2002). A study focused on event-related potentials (ERPs), an N2 component reflecting inhibitory control, showed that the amplitude of the No-go-N2 component increased as the relative frequency of Go *versus* No-go trials increased (Nieuwenhuis et al., 2003). To assess the effect of response history on cognitive control more directly, Freitas et al. (2007) conducted the Go/No-go trial immediately after the selective attention trial to investigate whether the response inhibition to No-go stimulus is affected by the response to the same stimulus in selective attention trials, that is, to see whether the stimulus was selected or ignored. The results of their study showed that the amplitude of the No-go-P3 component was enhanced when the stimulus was selected in the preceding selective attention trial; No-go-P3 was diminished when the stimulus was ignored.

However, most studies have focused on the effect of immediate action history, which is formed directly before the subsequent response in a laboratory environment. Additionally, these studies assessed the previous response effect by using the same type of responses, such as pro- or anti-saccades (Cherkasova et al., 2002; Manoach et al., 2007), and by selecting or ignoring responses (Freitas et al., 2007). No study has investigated the “long-term” response history (i.e., those that were not formed immediately before the subsequent behavior in a laboratory environment), with different type of responses. Thus, it remains unclear whether behavior in daily life affects action history on subsequent response control. About half of all everyday human behavior is repeated in the same context (Wood et al., 2002), and thus, our daily life largely consists of habitual and repetitive behavior (for review, Wood and Rünger, 2016). It is important to elucidate whether habitual and repetitive behavior affects action history, just like immediate action history that was made *ad hoc* in the laboratory environment. From this point of view, we focused on repetitive or habitual behavior in daily life as action history. In order to investigate the effect of repeated behavior, it is desirable that it has a short cycle of repetition and is frequently repeated in daily life.

Consumer behavior is a typical behavior in daily life comprising repetitive habitual characteristics (Ji and Wood, 2007; Wood and Neal, 2009; Van’t Riet et al., 2011). Particularly, consumers’ goods purchasing behavior, such as food and drinks, has relatively short purchase cycles and is repetitive and habitual. In this sense, daily consumption behavior can be viewed as a response history accumulated over a long period of time. Additionally, modern consumer behavior is not only about the purchase and consumption of specific goods, but also about the use of specific services. One service used repetitively and habitually for a particular brand is social networking sites. Thus, we focused on consumer behavior, especially the consumption of food or drinks, and the use of SNS.

It is reasonable to assume that the accumulated history of response to a particular stimulus formed in daily life would affect the inhibitory control to that stimulus in laboratory tasks. Frequently choosing or using specific products and services would establish habitual and prepotent behavior, so it would be difficult to ignore these stimuli and withhold a prepotent response. In order to test this prediction, we used the inhibitory-related ERPs observed during the Go/No-go task, as in previous studies. In the Go/No-go task, participants respond to a particular stimulus (Go trials) and withhold responses to another stimulus (No-go trials). ERPs recorded in the Go/No-go task reflect the rapid neural components of inhibitory control (Jodo and Kayama, 1992; Falkenstein et al., 1999). There are two representative ERP components reflecting inhibitory processes: No-go-N2 and No-go-P3. To investigate these components, analysis is time-locked to the presentation of No-go-stimuli. In many cases, No-go-N2 was recorded from the anterior field showing a negative peak at approximately 270ms. On the other hand, No-go-P3 was recorded from the parietal or posterior field showing a positive peak after 300ms (Falkenstein et al., 1999). Previous research has shown that the level of conflict monitoring is reflected as an amplitude of the No-go-N2 component, whereas the No-go-P3 component reflects the effort for motor inhibition (Liddle et al., 2001; Donkers and Van Boxtel, 2004; Lavric et al., 2004; Enriquez-Geppert et al., 2010; Kropotov et al., 2011). Through the analysis of these ERP components, it may be possible to objectively evaluate the habitual and repetitive nature of purchasing behavior as a response history.

Based on the above data, we conducted a Go/No-go task in the current study, using products with different purchase frequencies as stimuli. We aimed to elucidate the influence of “long-term” behavioral history formed in daily life by examining whether the No-go-related ERP components changed depending on the difference in the purchase frequency of stimuli. We hypothesized that withholding responses to the frequently chosen and purchased stimuli in participants’ daily lives would elicit greater No-go-related neural activity and show a weaker performance for action inhibition. Contrarily, withholding responses to infrequently chosen and purchased stimuli in daily life was predicted to elicit diminished No-go-related neural activity and intact task performance. To assess these hypotheses, we conducted a Go/No-go task in which the stimuli comprised real-life products, and recorded the ERP related to inhibitory control, that is, No-go-N2 and No-go-P3.

## Materials and Methods

### Participants

To select stimuli for the experiment, a preliminary survey was conducted with 192 candidates. Of these, 24 healthy right-handed adults (9 men and 15 women) satisfied the criteria of stimulus selection and participated in the present study (*M_age_* =27.8years, *SD* =7.2). All participants had a normal or corrected-to-normal vision. The study was approved by the Keio University Research Ethics Committee (No. 15039) and performed in accordance with the Declaration of Helsinki. All participants provided written informed consent prior to their participation in the electroencephalogram (EEG) recording experiment. Two participants were excluded *a posteriori* from the analysis, including behavioral data, because of the failure of peak detection during the ERP analysis. Therefore, the final sample comprised 22 participants.

### Stimuli

In order to select the stimuli for the Go and No-go trials, each participant responded to a question about the frequency of purchase or use of products and services. There were five categories of products: chocolates, carbonated drinks, beer, instant noodles, and SNS. Each category included five items; thus, participants evaluated the frequency of purchase or use of 25 products. The items used in each of the five categories include, for example, ‘Meiji Milk Chocolate (Meiji Holdings Co., Ltd.)’, ‘Mitsuya Cider (Asahi Soft Drinks Co., Ltd.)’, ‘YEBISU Beer (Sapporo Breweries Ltd.)’, ‘Cup Noodle (Nissin Food Products Co., Ltd.)’, and ‘Twitter (Twitter, Inc.)’, respectively. Each of these items is available in Japan. Participants were presented with the following question, “Having the opportunity to purchase and use each category of goods and services, how often do you purchase and use each item?” Participants reported the frequency of purchase and use on a 5-point scale, from 5 (*“Almost certainly buy or use it”*) to 1 (*“Hardly ever buy or use it”*).

Goods chosen by the participants as middle purchase frequency (i.e., 3) were used as Go stimuli, and those with high (5 or 4) or low (2 or 1) purchase frequency were used as No-go stimuli. These three stimuli were selected from the same product category for each participant. In other words, for each participant, no stimulus selected from a different category was presented. Only those who met these criteria, that is, those who had three items with different purchase frequencies in the same category, were able to join subsequent experiments. If there were items with the same rating, one of them was randomly selected as the stimulus.

The breakdown of the adopted categories among the participants, excluding the two participants who later failed in ERP detection and were consequently excluded from the analysis, was as follows: chocolates for four participants, carbonated drinks for two participants, beer for four participants, instant noodles for four participants, and SNS for eight participants.

The stimuli were presented in the form of pictures, (see author’s note) and the size of the pictures varied among the stimuli because they were real-life products and had very different packaging. However, the visual angle of the pictures was maintained at approximately 4–5°.

### Design and Procedure

Prior to the experiment, a preliminary survey was conducted on a different day. Participants responded to the purchase frequency survey to select stimuli for the subsequent experiment. Only those who met the aforementioned criteria of stimuli selection participated in subsequent EEG recording experiments after informed consent was obtained.

**Figure 1:**
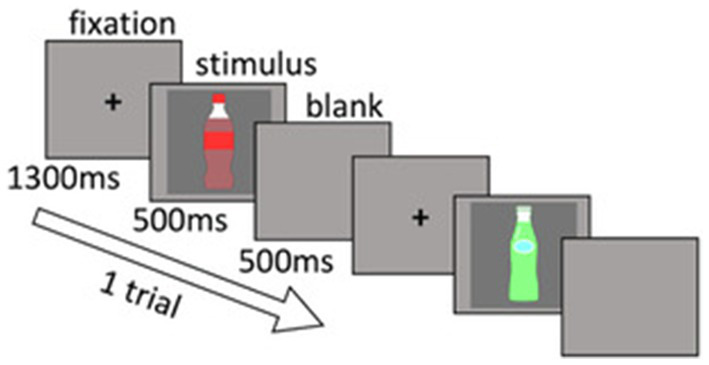
Go/No-go task structure. Each trial was composed of fixation, stimulus, and a blank screen. Participants were asked to respond with a button press as soon as possible when the Go stimulus was presented and to withhold responses when the No-go stimulus was presented. Each session was composed of 140 Go trials and 60 No-go trials. Six sessions were conducted in total. The stimuli of No-go trials were alternately switched between high and low purchase frequency in every session. In order not to infringe copyrights and trademarks, the two product images of the stimuli in this figure are replaced with dummies.

Each trial was composed of three parts: first, a fixation of 1,300ms; followed by the presentation of the stimulus (i.e., photographs of the product; see Figure 1) for 500ms; and lastly, the presentation of a blank screen for 500ms. Six sessions were conducted in total, with each session consisting of 140 Go trials and 60 No-go trials. The stimuli of the No-go trials were alternately switched between high and low purchase frequency in every session. For example, if the participants were exposed to a high purchase frequency product as a No-go stimulus in the first session, they were exposed to a low purchase frequency product as a No-go stimulus in the next session. Across all sessions, the middle purchase frequency product was the Go stimulus.

Participants were asked to respond with a button press as soon as possible when the Go stimulus was presented and to withhold responses when the No-go stimulus was presented. To respond, every participant used the index finger of their right hand. The participants completed 10 practice trials prior to the first session. Stimulus presentation and response recording were done using Presentation Ver. 12.1 (Neurobehavioral Systems).

### Electrophysiological Recording and Analysis

The EEG was recorded using NetStation 5.3.0.1 with a 65-channel HydroCel Geodesic Sensor Net (Electrical Geodesics, Inc.). The physical reference while recording was placed at the vertex (Cz) electrode, but the data were later re-referenced to the common average in pre-processing. The sampling rate was 500Hz, and electrode impedance was maintained below 50kΩ. Data were preprocessed and analyzed using EMSE 5.5.2 (Cortech Solutions, Inc.). We applied a 0.5–30Hz band-pass filter and a 50Hz notch filter. ERPs were computed and time-locked from −100ms to +500ms after the onset of the stimuli and baseline-corrected using a 100ms window before the stimulus. Epochs, including an EEG signal, exceeding ±100μV were excluded from the average. On average, 93.7% of the total number of epochs remained. The No-go-N2 component was calculated as the peak amplitude between 200 and 340ms. No-go-P3 was calculated as the mean amplitude between 330 and 470ms. ERPs were calculated only for successful No-go trials, that is, trials in which participants successfully withheld their responses. Two participants were rejected from the overall analysis because of the failure of ERP peak detection.

Many previous studies examining No-go-related ERPs have used electrodes near the top of the head, especially in the midline such as Fz, Cz, and Oz (Falkenstein et al., 1999; Donkers and Van Boxtel, 2004; Enriquez-Geppert et al., 2010; Kropotov et al., 2011, 2017). In the present study, after visually inspecting the topographical distribution of ERPs, Cz electrode was located in the center of relevant ERPs topographic mapping and was thus used in the subsequent analyses.

## Results

### Behavioral Data

**Figure 2:**
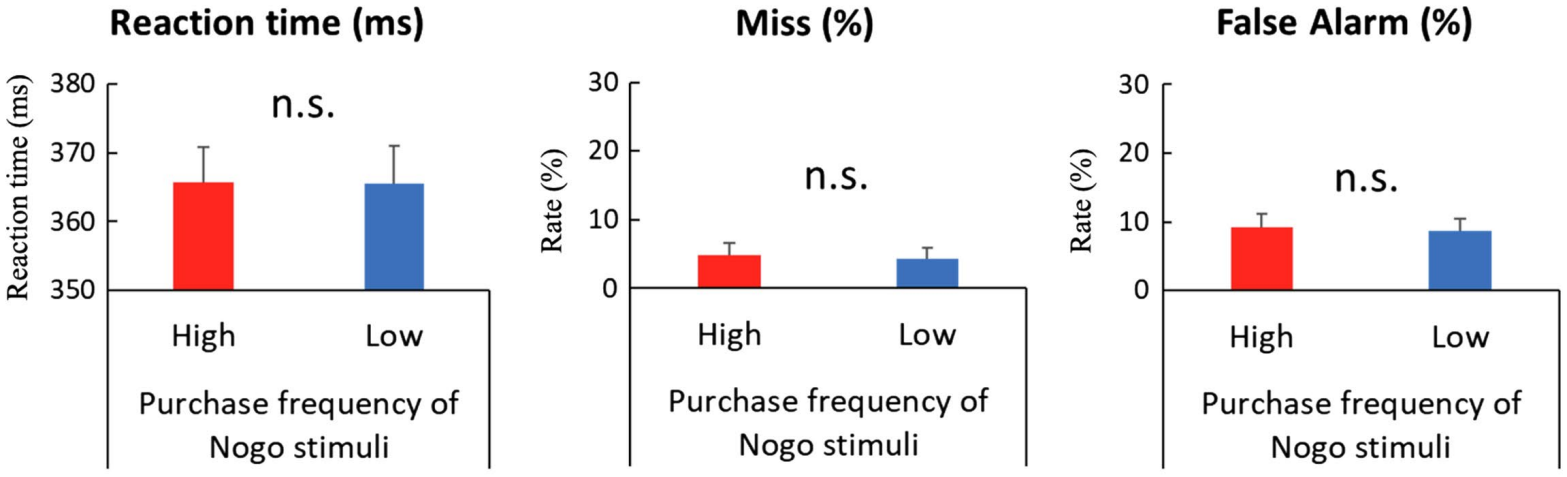
Comparisons of behavioral performance between high and low purchase frequency of No-go stimuli in Go/No-go task. Left: Reaction time of Go trial. Middle: Miss rate of Go trial. Right: False alarm rate of No-go trial. Error bars in all panels represent standard error of the mean. None of the differences were significant. n.s.=not significant.

In the entire analysis, we did not consider the qualitative differences of each product category but treated them as equal and analyzed behavioral performances and EEG data according to differences in stimulus conditions based on the frequency of purchase and use. To examine the effect of the No-go stimuli’s purchase frequency on behavioral data, we calculated the reaction time and miss (omission error) rate of the Go trial, false alarm (commission error) rate of No-go trial, and deployed paired *t*-tests to compare high and low purchase frequency conditions. In order to analyze the reaction times of Go trials, we first excluded trials with a reaction time of less than 100ms as outliers, and trials where participants responded after the Go stimulus had disappeared. For the remaining trials, trials with more than ±3 SD from the mean reaction time of each participant were excluded; the remaining trials were categorized as correct Go response (hit) or incorrect Go response (miss). Since the Go stimulus is always the same regardless of the purchase frequency condition of the No-go stimulus, we analyzed the behavioral data of Go trials between sessions in which the stimulus with high purchase frequency was presented and sessions in which the stimulus with low purchase frequency was presented for the No-go stimulus. As a result, the reaction times of the correct Go response showed no significant differences between purchase frequency [365.46ms and 366ms for high and low purchase frequency conditions, respectively; *t* (21)=−0.18, *p*=0.86]. The miss rate of the Go trial showed no significant differences between purchase frequency [4.76 and 4.29% for high and low purchase frequency conditions, respectively; *t* (21)=1.14, *p*=0.27]. Since the same stimuli were continuously used in the Go trials regardless of the purchase frequency conditions of the No-go stimuli, it is reasonable that behavioral performance in the Go trials exhibited no discernible difference. The false alarm rates of the No-go trial, calculated as the percentage of trials in which participants failed to withhold responses until the next trial began, had no discernible differences between purchase frequency [9.24 and 8.66% for high and low purchase frequency conditions, respectively; *t* (21)=0.38, *p*=0.71]. In summary, there was no significant difference in any of the behavioral indices (Figure 2).

### ERP Data

**Figure 3:**
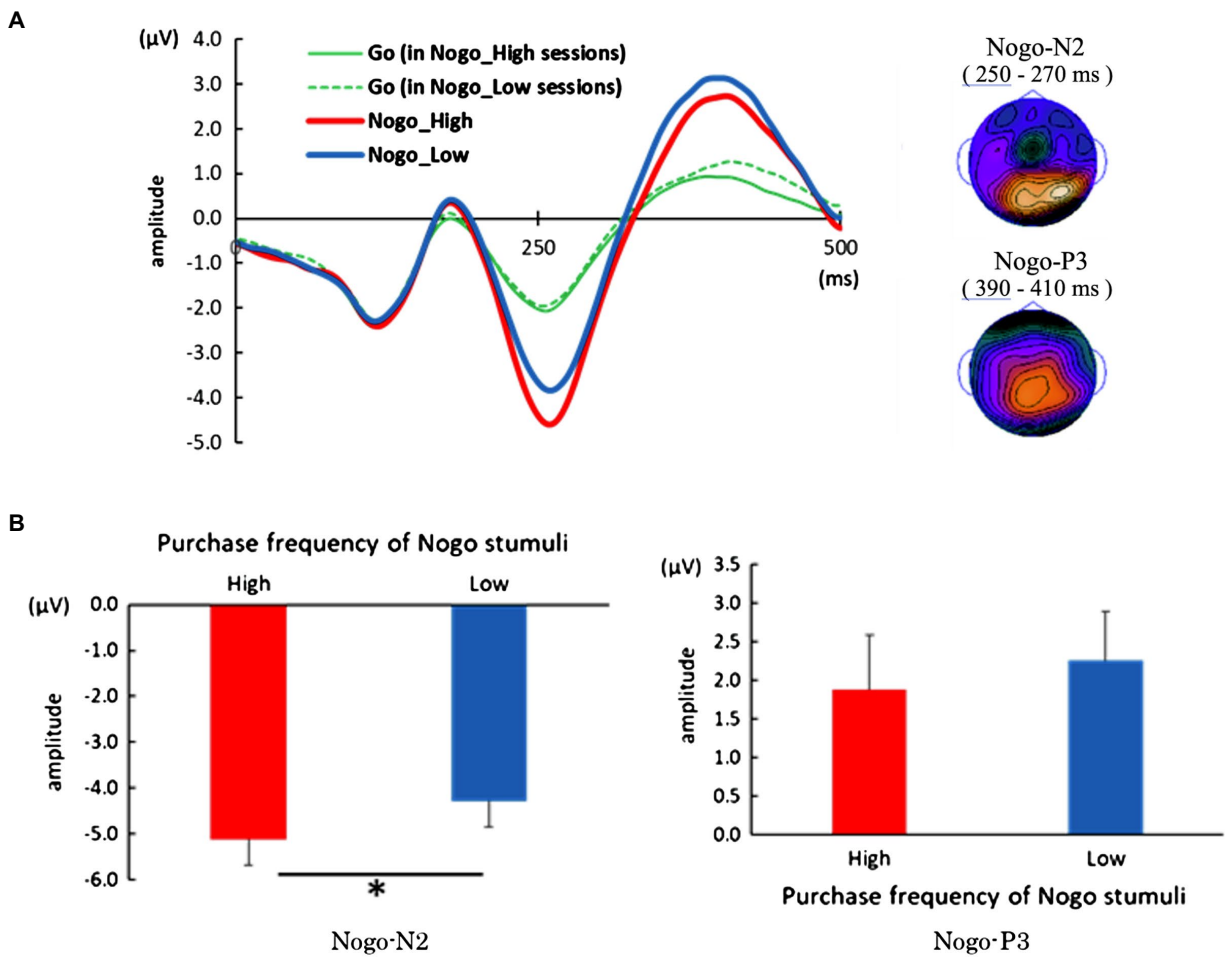
Event-related potential (ERP) analysis of Go/No-go task. **(A)** Left: An averaged waveform of ERP which was time-locked on the presentation of stimuli. Typical No-go-N2 and No-go-P3 components can be seen. Right: Topographical distribution of No-go-N2 and No-go-P3 components. A pronounced negative and positive distribution can be observed in the parietal area at the time corresponding to the No-go-N2 and No-go-P3 components, respectively. **(B)** Results of paired *t*-test of the peak amplitude of No-go-N2 (left panel) and No-go-P3 (right panel). No-go trials with high purchase frequency products elicited significantly greater No-go-N2 amplitudes. Error bars in both panels represent standard error of the mean. *Significant on *p*<0.05.

A pronounced negative and positive distribution can be observed in the parietal area at the time corresponding to the No-go-N2 and No-go-P3 components, respectively, and we observed a salient No-go-N2 and slightly sloping No-go-P3 on electrode Cz (Figure 3A). We calculated the peak amplitude between 200 and 340ms, and 330 and 470ms as No-go-N2 and No-go-P3, respectively, in the No-go trial with high purchase frequency product and the trial with low purchase frequency product (Figure 3B). We conducted a paired *t*-test to evaluate the difference between the high and low purchase frequency of No-go stimuli in the peak amplitude of each component. As a result, there was a significant difference of peak amplitude only in the No-go-N2 component between the high and low purchase frequency [*t* (21)=2.51, *p*=0.02, Hedge’s *g*=0.53]. No-go trials with high purchase frequency products elicited greater No-go-N2 amplitudes. On the other hand, there was no significant difference in the No-go-P3 component [*t* (21)=0.82, *p*=0.42].

## Discussion

In the current study, we investigated the effect of response history formed in daily life, rather than short-term response history formed *ad hoc* in the laboratory. We treated the frequency of participants’ daily behavior of buying and using products as response history and investigated whether the difference in frequency, that is, the difference in response history, modulated the response control in a Go/No-go task.

Our results showed that the No-go trial with frequently purchased products elicited a greater No-go-N2 component than those with less frequently purchased products. It is important to note that no conditioning or learning took place during the course of the experiment. The main difference was seen in how often participants buy or use products in their daily lives. Nonetheless, there was a significant difference in the amplitude of No-go-N2 depending on the frequency of purchase or use in daily life. Additionally, participants’ behavioral responses to the stimuli in response history were concrete and complex everyday behaviors, such as purchasing products and using SNS, but the Go/No-go task was a simple action of pressing a button or not pressing it.

The No-go-N2 component is considered to reflect the response conflict in cognitive control (Liddle et al., 2001; Donkers and Van Boxtel, 2004; Lavric et al., 2004; Enriquez-Geppert et al., 2010; Kropotov et al., 2011). Previous research on the source localization of No-go-related ERP components showed that the source of the No-go-N2 component was the medial prefrontal cortex, especially around the anterior cingulate cortex (ACC; Bokura et al., 2001; Braver et al., 2001; Leung and Cai, 2007; Chmielewski and Beste, 2017). A number of studies have shown that the ACC is closely related to conflict monitoring (Van Veen et al., 2001; Van Veen and Carter, 2002; Botvinick et al., 2004; Kerns et al., 2004). Our findings thus suggest that withholding responses to frequently purchased stimuli evoked a stronger conflict. In other words, the response history of particular stimuli formed in daily consumer behavior elicited greater conflict when it became necessary to ignore those stimuli. It is considered that repeated purchase behavior for a product forms a strong stimulus–response association, and the stimulus tends to trigger automatic response, resulting in a stronger conflict in suppressing such automatic reactions. A similar mechanism has been pointed out in research conducted on habitual behaviors. Behavior performed repeatedly becomes habitual and is then triggered by contextual cues and guided by an automated process, rather than an elaborate cognitive process (Aarts et al., 1998; Neal et al., 2012). As a result, a greater response conflict will arise when withholding such automatic and habitual responses.

There is another possible interpretation for the observed No-go-N2 conditional difference. We assumed that frequently purchased stimuli elicited stronger approach motivation for participants; thus, greater conflict was evoked when resisting approach motivation and withholding response to the stimuli, resulting in a greater amplitude of No-go-N2. Since frequent purchases indicate that a positive attitude toward the product was formed to some extent, we can assume that the response history of frequent purchasing is linked to approach motivation. Therefore, we can predict that rarely purchased products have no or relatively weak approach motivation and that participants face fewer conflicts during withdrawal behavior, resulting in a significant difference in No-go-N2 amplitude between frequently purchased stimuli and rarely purchased stimuli. It is a reasonable interpretation in light of another result of current study, which showed that the effect of reaction history occurs even when the type of the response in the past is different from that required in the present. A similar structure has been found in substance abuse patients’ inhibitory control studies. Substance abuse patients exhibit a strong attentional bias to substance-related stimuli and often have difficulty inhibiting responses to drug-related stimuli (Cox et al., 2002; Kaufman et al., 2003; Marissen et al., 2006; Field et al., 2009; Luijten et al., 2011, 2014; Wilcockson and Pothos, 2015). One of the theories explaining these traits of substance abuse patients states that a strong approach motivation for substance easily captures one’s attention, making it difficult to inhibit response to drug-related stimuli.

In the present study, there was a large gap between the frequency of presentation of Go and No-go stimuli. Therefore, it can be considered that the N2 components in the current study may reflect the response to rare (deviant) stimuli as seen in the typical oddball paradigm. In fact, the general tendency observed in the Go/No-go task is that the lower the ratio of No-go trials presented, the larger is the No-go-N2 amplitude (Bruin and Wijers, 2002; Kubo et al., 2021). However, some previous studies have reported that No-go-N2 can be observed even when the probability of presentation of Go and No-go trials is equal (Jodo and Kayama, 1992; Nieuwenhuis et al., 2003; Lavric et al., 2004; Pandey et al., 2012). Thus, in the context of inhibitory control task, it is reasonable to consider that the No-go-N2 component mainly reflects a demand of inhibitory control or the degree of conflict monitoring rather than an oddball-like novelty effect. In addition, the probability of high purchase frequency No-go stimuli was equivalent to that of low purchase frequency No-go stimuli; hence, it may be reasonable to conclude that the difference in the No-go-N2 amplitude found between high and low purchase frequencies was not caused by the differences in presentation probability. An oddball-like N2 for rare stimuli may have overlapped, but even if that were the case, it does not affect the conclusion of the main results of the present study.

The large difference in the probability of presentation across stimuli may have also affected the familiarity of the stimuli within the experiment. In this regard, since the number of No-go trials was identical between high and low purchase frequency stimuli, it is not reasonable to assume that the familiarity bias among No-go stimuli occurred during the course of the experiment. However, it is natural to assume that the products with high purchase frequency had a higher degree of familiarity from the beginning. Thus, it can be inferred that the higher familiarity of No-go stimuli with high purchase frequency caused the greater No-go-N2 amplitude. If it is assumed that high familiarity directly enhances the N2 amplitude, then N2 should have been the largest in the Go trials, where the number of presentations was overwhelmingly large; however, this was not the case. Instead, a more likely explanation may that withholding response to highly familiar stimuli causes greater conflict and requires more effortful cognitive control. There is evidence of dissociation in the neural mechanism underlying response conflict and familiarity conflict using functional magnetic resonance imaging (Nelson et al., 2003); these researchers showed that the ACC is involved in response conflict, whereas the left inferior frontal gyrus is involved in familiarity conflict. The association between response conflict and the ACC is consistent with the findings from previous EEG/ERP research; thus, using functional imaging will be an effective approach to address this question. Since the present study was not devised to control the familiarity of the stimuli, direct manipulation of familiarity remains an issue for future study. Further research is required to elucidate the detailed mechanisms of enhanced No-go-N2 observed in the present study.

In contrast to No-go-N2, there was no significant difference in the purchase frequency in No-go-P3. The No-go-P3 component is considered to reflect inhibitory effort or the motor processing of inhibitory control (Donkers and Van Boxtel, 2004; Groom and Cragg, 2015). In fact, previous research suggested that the source of the No-go-P3 component is the supplementary motor cortex (Bokura et al., 2001; Kropotov et al., 2011). Therefore, it is reasonable that No-go-P3 is less sensitive to purchase frequency and more sensitive to task difficulty and performance.

As with the No-go-P3 component, there was no significant difference in any of the behavioral performance measures. Although inconsistent with previous research, a straightforward interpretation of the results suggests that response history does not affect task performance. However, it must be mentioned that the task we used in the present study was low in difficulty. In general, it is said that the longer the preparation, the more accurate the response control. In the current study, we set a relatively long fixation screen. Additionally, there was only one Go stimulus and one No-go stimulus in each session; therefore, it seems that it was not difficult to select a correct response. Thus, it is possible that the low task difficulty may have contaminated the inherent impact of the difference in purchase frequency.

Unlike previous studies on response history, conflicting behaviors in the present study were completely different. A previous study suggested that greater response control is required when current cue–response associations are incompatible with past associations (Freitas et al., 2007). Applying a straightforward interpretation of prior research, the direct incompatibility of the required motor action between the preceding and current responses would evoke greater response conflict, thereby resulting in greater No-go-N2 amplitude. On the other hand, in our study, the preceding stimulus–response association decided whether to repeat a purchase behavior or not, while the Go/No-go task required the participants to execute or withhold the button press response. Therefore, the cue–response association in action history and the stimulus–response association required to perform the task were different regardless of the condition of purchase frequency. This means that all conditions were equivalent in the sense that participants were required to make a response that was contrary to the preformed stimulus–response association. Nevertheless, the No-go trials with high purchase frequency products elicited a stronger effect of response conflict as No-go-N2; we should consider an explanation other than the previous research that used the same type of behavior in past and present responses, such as differences in the direction of response (Cherkasova et al., 2002; Manoach et al., 2007), and the presence or absence of the response (Freitas et al., 2007).

Our study has some limitations that should be pointed out. First, it remains unclear whether the strong response conflict arises from the high frequency of consumption or from strong approach motivation, such as a high degree of liking for the stimuli, or higher familiarity of the stimuli. Second, we were unable to provide direct evidence for the different roles of No-go-N2 and No-go-P3 with respect to response history. Approach motivation might consist not only of automatic and habitual tendency to respond but also of a positive attitude or preference without a substantial response history. In the present study, we only surveyed and controlled the frequency of purchase, and not the preference or the familiarity of the stimulus. One of the future prospects for resolving these limitations is to examine the changes in No-go-N2 and No-go-P3 as a function of response history, approach motivation, and familiarity. More detailed studies in this area are required. Third, because we were not able to use participants’ actual purchase records and hence used self-reporting questionnaire, we could not use precise data of frequency and period of purchasing behavior. A possible solution to this limitation is to use a product group that allows the use of point-of-sales data tied to the consumption behavior of each individual.

Our findings highlight that the amplitude of the No-go-N2 component showed sensitivity to the purchase frequency of stimuli. Thus, from an applied psychophysiological perspective, the No-go-N2 component could be utilized as a predictive value of purchase frequency or potential preference of products. The fact that the accumulation of daily purchasing behavior influenced the response suppression in the laboratory as a response history indicates that behavioral tendencies in daily life can be verified in laboratory experiments. The degree of robustness of daily habitual and repetitive behaviors and decision-making, such as consumption behavior, can be potentially indexed by performance and inhibition-related ERP in the Go/No-go task. Other studies have used EEG and ERP as indicators of consumer behavior (Ohme et al., 2010; Khushaba et al., 2013; Yımaz et al., 2014; Telpaz et al., 2015), thus supporting the usefulness of EEG and ERP as analytical tools and indicators of consumer behavior.

## Conclusion

In conclusion, the current study showed that the response history has a long-term impact and that previous trial effects occur even during different behaviors, such as consumer behavior in daily life and response control in an experimental task. Additionally, the present study implies that the mechanism by which past response history has an influence on present behavior is associated with habitual behavior, consumer behavior, and substance abuse behavior. Further studies must integrate these findings based on more generalized mechanisms that control human behavior.

## Data Availability Statement

The raw data supporting the conclusions of this article will be made available by the authors, without undue reservation.

## Ethics Statement

The studies involving human participants were reviewed and approved by the Keio University Research Ethics Committee. The patients/participants provided their written informed consent to participate in this study.

## Author’s Note

The actual stimuli images used in the experiment are not included in this article to avoid copyright and trademark infringement. The images will be made available by the Corresponding Author, without undue reservation.

## Author Contributions

KT: experimental design, data collection, data analysis, interpretation of results, and preparation of manuscript. MS: data collection, data analysis, and interpretation of results. YT and SU: experimental design of behavioral experiment and interpretation of results. All authors contributed to the article and approved the submitted version.

## Funding

This research was supported by a Grant-in-Aid for JSPS Fellows to KT (No. JP18J15130) and a Grant-in-Aid for Scientific Research on Innovative Areas to SU (No. JP18H05525) from the Ministry of Education, Culture, Sports, Science, and Technology (MEXT).

## Conflict of Interest

The authors declare that the research was conducted in the absence of any commercial or financial relationships that could be construed as a potential conflict of interest.

## Publisher’s Note

All claims expressed in this article are solely those of the authors and do not necessarily represent those of their affiliated organizations, or those of the publisher, the editors and the reviewers. Any product that may be evaluated in this article, or claim that may be made by its manufacturer, is not guaranteed or endorsed by the publisher.

## References

[ref1] Aarts H. Verplanken B. Van Knippenberg A. (1998). Predicting behavior from actions in the past: repeated decision making or a matter of habit? J. Appl. Soc. Psychol. 28, 1355–1374. doi: 10.1111/j.1559-1816.1998.tb01681.x

[ref2] Barton J. J. S. Greenzang C. Hefter R. Edelman J. Manoach D. S. (2006). Switching, plasticity, and prediction in a saccadic task-switch paradigm. Exp. Brain Res. 168, 76–87. doi: 10.1007/s00221-005-0091-1, PMID: 16096781

[ref3] Bokura H. Yamaguchi S. Kobayashi S. (2001). Electrophysiological correlates for response inhibition in a Go/NoGo task. Clin. Neurophysiol. 112, 2224–2232. doi: 10.1016/S1388-2457(01)00691-5, PMID: 11738192

[ref4] Botvinick M. M. Cohen J. D. Carter C. S. (2004). Conflict monitoring and anterior cingulate cortex: an update. Trends Cogn. Sci. 8, 539–546. doi: 10.1016/j.tics.2004.10.003, PMID: 15556023

[ref5] Braver T. S. Barch D. M. Gray J. R. Molfese D. L. Snyder A. (2001). Anterior cingulate cortex and response conflict: effects of frequency, inhibition and errors. Cereb. Cortex 11, 825–836. doi: 10.1093/cercor/11.9.825, PMID: 11532888

[ref6] Bruin K. J. Wijers A. A. (2002). Inhibition, response mode, and stimulus probability: a comparative event-related potential study. Clin. Neurophysiol. 113, 1172–1182. doi: 10.1016/S1388-2457(02)00141-4, PMID: 12088714

[ref7] Cherkasova M. V. Manoach D. S. Intriligator J. M. Barton J. J. (2002). Antisaccades and task-switching: interactions in controlled processing. Exp. Brain Res. 144, 528–537. doi: 10.1007/s00221-002-1075-z, PMID: 12037637

[ref8] Chmielewski W. X. Beste C. (2017). Testing interactive effects of automatic and conflict control processes during response inhibition – A system neurophysiological study. NeuroImage 146, 1149–1156. doi: 10.1016/j.neuroimage.2016.10.015, PMID: 27742599

[ref9] Ciardo F. Ricciardelli P. Iani C. (2019). Trial-by-trial modulations in the orienting of attention elicited by gaze and arrow cues. Q. J. Exp. Psychol. 72, 543–556. doi: 10.1177/1747021818769588 29589789

[ref10] Cox W. M. Hogan L. M. Kristian M. R. Race J. H. (2002). Alcohol attentional bias as a predictor of alcohol abusers’ treatment outcome. Drug Alcohol Depend. 68, 237–243. doi: 10.1016/S0376-8716(02)00219-3, PMID: 12393218

[ref11] Donkers F. C. L. Van Boxtel G. J. M. (2004). The N2 in go/no-go tasks reflects conflict monitoring not response inhibition. Brain Cogn. 56, 165–176. doi: 10.1016/j.bandc.2004.04.005, PMID: 15518933

[ref12] Durston S. Thomas K. M. Yang Y. Uluǧ A. M. Zimmerman R. D. Casey B. J. (2002). A neural basis for the development of inhibitory control. Dev. Sci. 5, 9–16. doi: 10.1111/1467-7687.00235

[ref13] Enriquez-Geppert S. Konrad C. Pantev C. Huster R. J. (2010). Conflict and inhibition differentially affect the N200/P300 complex in a combined go/nogo and stop-signal task. NeuroImage 51, 877–887. doi: 10.1016/j.neuroimage.2010.02.043, PMID: 20188191

[ref14] Falkenstein M. Hoormann J. Hohnsbein J. (1999). ERP components in Go/Nogo tasks and their relation to inhibition. Acta Psychol. 101, 267–291. doi: 10.1016/S0001-6918(99)00008-6 10344188

[ref15] Fecteau J. H. Au C. Armstrong I. T. Munoz D. P. (2004). Sensory biases produce alternation advantage found in sequential saccadic eye movement tasks. Exp. Brain Res. 159, 84–91. doi: 10.1007/s00221-004-1935-9, PMID: 15243727

[ref16] Fecteau J. H. Munoz D. P. (2003). Exploring the consequences of the previous trial. Nat. Rev. Neurosci. 4, 435–443. doi: 10.1038/nrn1114, PMID: 12778116

[ref17] Field M. Munafò M. R. Franken I. H. A. (2009). A meta-analytic investigation of the relationship between attentional bias and subjective craving in substance abuse. Psychol. Bull. 135, 589–607. doi: 10.1037/a0015843, PMID: 19586163 PMC2999821

[ref18] Freitas A. L. Azizian A. Leung H. C. Squires N. K. (2007). Resisting recently acted-on cues: compatibility of Go/NoGo responses to response history modulates (frontal P3) event-related potentials. Psychophysiology 44, 2–10. doi: 10.1111/j.1469-8986.2006.00484.x, PMID: 17241136

[ref19] Groom M. J. Cragg L. (2015). Differential modulation of the N2 and P3 event-related potentials by response conflict and inhibition. Brain Cogn. 97, 1–9. doi: 10.1016/j.bandc.2015.04.004, PMID: 25955278

[ref20] Ji M. F. Wood W. (2007). Purchase and consumption habits: not necessarily what you intend. J. Consum. Psychol. 17, 261–276. doi: 10.1016/S1057-7408(07)70037-2

[ref21] Jodo E. Kayama Y. (1992). Relation of a negative ERP component to response inhibition in a Go/No-go task. Electroencephalogr. Clin. Neurophysiol. 82, 477–482. doi: 10.1016/0013-4694(92)90054-L, PMID: 1375556

[ref22] Kaufman J. N. Ross T. J. Stein E. A. Garavan H. (2003). Cingulate hypoactivity in cocaine users during a GO-NOGO task as revealed by event-related functional magnetic resonance imaging. J. Neurosci. 23, 7839–7843. doi: 10.1523/JNEUROSCI.23-21-07839.2003, PMID: 12944513 PMC6740597

[ref23] Kerns J. G. Cohen J. D. MacDonald A. W. Cho R. Y. Stenger V. A. Carter C. S. (2004). Anterior cingulate conflict monitoring and adjustments in control. Science 303, 1023–1026. doi: 10.1126/science.1089910, PMID: 14963333

[ref24] Khushaba R. N. Wise C. Kodagoda S. Louviere J. Kahn B. E. Townsend C. (2013). Consumer neuroscience: assessing the brain response to marketing stimuli using electroencephalogram (EEG) and eye tracking. Expert Syst. Appl. 40, 3803–3812. doi: 10.1016/j.eswa.2012.12.095

[ref25] Kropotov J. D. Ponomarev V. A. Hollup S. Mueller A. (2011). Dissociating action inhibition, conflict monitoring and sensory mismatch into independent components of event related potentials in GO/NOGO task. NeuroImage 57, 565–575. doi: 10.1016/j.neuroimage.2011.04.060, PMID: 21571079

[ref26] Kropotov J. D. Ponomarev V. A. Pronina M. Jäncke L. (2017). Functional indexes of reactive cognitive control: ERPs in cued go/no-go tasks. Psychophysiology 54, 1899–1915. doi: 10.1111/psyp.12960, PMID: 28771747

[ref27] Kubo N. Watanabe T. Chen X. Matsumoto T. Yunoki K. Kuwabara T.. (2021). The effect of prior knowledge of color on behavioral responses and event-related potentials during Go/No-go task. Front. Hum. Neurosci. 15, 1–12. doi: 10.3389/fnhum.2021.674964 PMC8222725 34177494

[ref28] Lavric A. Pizzagalli D. A. Forstmeier S. (2004). When ‘go’ and ‘nogo’ are equally frequent: ERP components and cortical tomography. Eur. J. Neurosci. 20, 2483–2488. doi: 10.1111/j.1460-9568.2004.03683.x, PMID: 15525290

[ref29] Leung H. C. Cai W. (2007). Common and differential ventrolateral prefrontal activity during inhibition of hand and eye movements. J. Neurosci. 27, 9893–9900. doi: 10.1523/JNEUROSCI.2837-07.2007, PMID: 17855604 PMC6672638

[ref30] Liddle P. F. Kiehl K. A. Smith A. M. (2001). Event-related fMRI study of response inhibition. Hum. Brain Mapp. 12, 100–109. doi: 10.1002/1097-0193(200102)12:2<100::AID-HBM1007>3.0.CO;2-6, PMID: 11169874 PMC6871906

[ref31] Luijten M. Littel M. Franken I. H. A. (2011). Deficits in inhibitory control in smokers during a Go/NoGo task: An investigation using event-related brain potentials. PLoS One 6:e18898. doi: 10.1371/journal.pone.0018898, PMID: 21526125 PMC3081309

[ref32] Luijten M. Machielsen M. W. J. Veltman D. J. Hester R. de Haan L. Franken I. H. A. (2014). Systematic review of ERP and fMRI studies investigating inhibitory control and error processing in people with substance dependence and behavioural addictions. J. Psychiatry Neurosci. 39, 149–169. doi: 10.1503/jpn.130052, PMID: 24359877 PMC3997601

[ref33] Manoach D. S. Thakkar K. N. Cain M. S. Polli F. E. Edelman J. A. Fischl B.. (2007). Neural activity is modulated by trial history: a functional magnetic resonance imaging study of the effects of a previous antisaccade. J. Neurosci. 27, 1791–1798. doi: 10.1523/JNEUROSCI.3662-06.2007, PMID: 17301186 PMC6673726

[ref34] Marissen M. A. E. Franken I. H. A. Waters A. J. Blanken P. Van Den Brink W. Hendriks V. M. (2006). Attentional bias predicts heroin relapse following treatment. Addiction 101, 1306–1312. doi: 10.1111/j.1360-0443.2006.01498.x, PMID: 16911730

[ref35] Neal D. T. Wood W. Labrecque J. S. Lally P. (2012). How do habits guide behavior? Perceived and actual triggers of habits in daily life. J. Exp. Soc. Psychol. 48, 492–498. doi: 10.1016/j.jesp.2011.10.011

[ref36] Nelson J. K. Reuter-Lorenz P. A. Sylvester C. Y. C. Jonides J. Smith E. E. (2003). Dissociable neural mechanisms underlying response-based and familiarity-based conflict in working memory. Proc. Natl. Acad. Sci. U. S. A. 100, 11171–11175. doi: 10.1073/pnas.1334125100 12958206 PMC196946

[ref37] Nieuwenhuis S. Yeung N. van den Wildenberg W. Ridderinkhof K. R. (2003). Electrophysiological correlates of anterior cingulate function in a go/no-go task: effects of response conflict and trial type frequency. Cogn. Affect. Behav. Neurosci. 3, 17–26. doi: 10.3758/CABN.3.1.17, PMID: 12822595

[ref39] Ohme R. Reykowska D. Wiener D. Choromanska A. (2010). Application of frontal EEG asymmetry to advertising research. J. Econ. Psychol. 31, 785–793. doi: 10.1016/j.joep.2010.03.008

[ref40] Pandey A. K. Kamarajan C. Tang Y. Chorlian D. B. Roopesh B. N. Manz N.. (2012). Neurocognitive deficits in male alcoholics: An ERP/sLORETA analysis of the N2 component in an equal probability Go/NoGo task. Biol. Psychol. 89, 170–182. doi: 10.1016/j.biopsycho.2011.10.009, PMID: 22024409 PMC3245806

[ref41] Reuter E. M. Marinovic W. Welsh T. N. Carroll T. J. (2019). Increased preparation time reduces, but does not abolish, action history bias of saccadic eye movements. J. Neurophysiol. 121, 1478–1490. doi: 10.1152/jn.00512.2018, PMID: 30785812 PMC6485728

[ref42] Telpaz A. Webb R. Levy D. J. (2015). Using EEG to predict consumers’ future choices. J. Mark. Res. 52, 511–529. doi: 10.1509/jmr.13.0564

[ref43] Valyear K. F. Fitzpatrick A. M. Dundon N. M. (2019). Now and then: hand choice is influenced by recent action history. Psychon. Bull. Rev. 26, 305–314. doi: 10.3758/s13423-018-1510-1, PMID: 30039397 PMC6424939

[ref44] Van Veen V. Carter C. S. (2002). The anterior cingulate as a conflict monitor: fMRI and ERP studies. Physiol. Behav. 77, 477–482. doi: 10.1016/S0031-9384(02)00930-7, PMID: 12526986

[ref45] Van Veen V. Cohen J. D. Botvinick M. M. Stenger V. A. Carter C. S. (2001). Anterior cingulate cortex, conflict monitoring, and levels of processing. NeuroImage 14, 1302–1308. doi: 10.1006/nimg.2001.0923, PMID: 11707086

[ref46] Van’t Riet J. Sijtsema S. J. Dagevos H. de Bruijn G. J. (2011). The importance of habits in eating behaviour: an overview and recommendations for future research. Appetite 57, 585–596. doi: 10.1016/j.appet.2011.07.010 21816186

[ref47] Wilcockson T. D. W. Pothos E. M. (2015). Measuring inhibitory processes for alcohol-related attentional biases: introducing a novel attentional bias measure. Addict. Behav. 44, 88–93. doi: 10.1016/j.addbeh.2014.12.015, PMID: 25583563

[ref001] Witney A. G. Vetter P. Wolpert D. M. (2001). The influence of previous experience on predictive motor control. Neuroreport 12, 649–653. doi: 10.1097/00001756-200103260-00007, PMID: 11277557

[ref48] Wood W. Neal D. T. (2009). The habitual consumer. J. Consum. Psychol. 19, 579–592. doi: 10.1016/j.jcps.2009.08.003

[ref49] Wood W. Quinn J. M. Kashy D. A. (2002). Habits in everyday life: thought, emotion, and action. J. Pers. Soc. Psychol. 83, 1281–1297. doi: 10.1037/0022-3514.83.6.1281, PMID: 12500811

[ref50] Wood W. Rünger D. (2016). Psychology of habit. Annu. Rev. Psychol. 67, 289–314. doi: 10.1146/annurev-psych-122414-033417, PMID: 26361052

[ref51] Yımaz B. Korkmaz S. Arslan D. B. Güngör E. Asyalı M. H. (2014). Like/dislike analysis using EEG: determination of most discriminative channels and frequencies. Comput. Methods Prog. Biomed. 113, 705–713. doi: 10.1016/j.cmpb.2013.11.010 24326336

